# Sonographic and CT imaging features of intestinal perforation from a pill and packing: A case report

**DOI:** 10.1097/MD.0000000000010427

**Published:** 2018-04-20

**Authors:** Ze-hui Gou, Yulan Peng, Kun Yang

**Affiliations:** a Department of Ultrasound; b Department of Gastrointestinal Surgery, West China Hospital, Sichuan University, Chengdu, China.

**Keywords:** appendix, computed tomography, CT, foreign body, ileum, intestinal perforation, sonographic, ultrasound, US

## Abstract

**Rationale:**

Sharp foreign bodies such as toothpicks or chicken bones can lead to intestinal perforation. Small intestinal perforation secondary to foreign body ingestion is usually manifested as an acute abdomen without a history of trauma. Here we describe the diagnosis and treatment of a case of small bowel perforation caused by an ingested pill and its outer packing.

**Patient concerns:**

An 84 years old male patient complained of right lower abdominal pain for 4 days and the pain was becoming progressively worse.

**Diagnoses:**

The patient, who has Alzheimer's disease, mistakenly took the pill (oxiracetam) without removing the outer packaging. This resulted in perforation of the small intestine.

**Interventions:**

During the ultrasound examination, the scanning physician discovered that the abnormal sonographic findings present could not be explained by the leading diagnosis of perforation of the small intestine at the time. This led the physician to suspect small bowel perforation secondary to a foreign body. The subsequent computerized tomography (CT) examination further confirmed the ultrasound findings.

**Outcomes:**

Emergency laparotomy was performed and the foreign body was removed. After the surgical procedure, the patient resumed anti-inflammatory treatment (Cefoxitin sodium 2000mg tid) and rehydration therapy (Sodium Chloride Solution 100mL tid).

**Lessons:**

Because ingestion of foreign bodies of this type is relatively rare, when patients cannot provide an accurate history, diagnosis can be quite difficult. In this paper, the imaging features associated with intestinal perforation secondary to foreign body ingestion on ultrasound and CT are described. This series of events demonstrate how imaging findings can guide and alter a clinician's decision-making.

## 1 Introduction

Sharp foreign bodies such as toothpicks or chicken bones can lead to intestinal perforation.^[1–3]^ Small intestinal perforation secondary to a foreign body is usually manifested as an acute abdomen without a history of trauma. These patients tend not to chew carefully, and therefore tend to be mostly young, toothless, mentally retarded, elderly, or have psychiatric symptoms.^[4]^ Small intestinal perforation caused by pill packaging as in this case is rare. Computerized tomography (CT) and ultrasound offer strong diagnostic accuracy in such a case. Patient history should prompt the use of these diagnostic methodologies in a certain subset of patients.

## 2 Patient information

An 84-year-old male patient was admitted to our hospital in July of this year. Three days before arrival at our hospital, the patient had been misdiagnosed with acute appendicitis at an outside hospital. Before admission, the patient was treated at the outside hospital with fasting and anti-inflammatory medications, which temporarily alleviated his pain. However, when the patient noted black tarry stools, he sought further emergency treatment in our department.

Afterward, the patient underwent emergency exploratory laparotomy. Ex-lap confirmed the findings on CT and ultrasound as ileal perforation secondary to a foreign body.

## 3 Clinical findings

On physical examination, the patient's abdomen was soft without right lower quadrant tenderness, rebound tenderness, muscle tension, or palpable mass.

## 4 Diagnostic assessment

**Figure 1:**
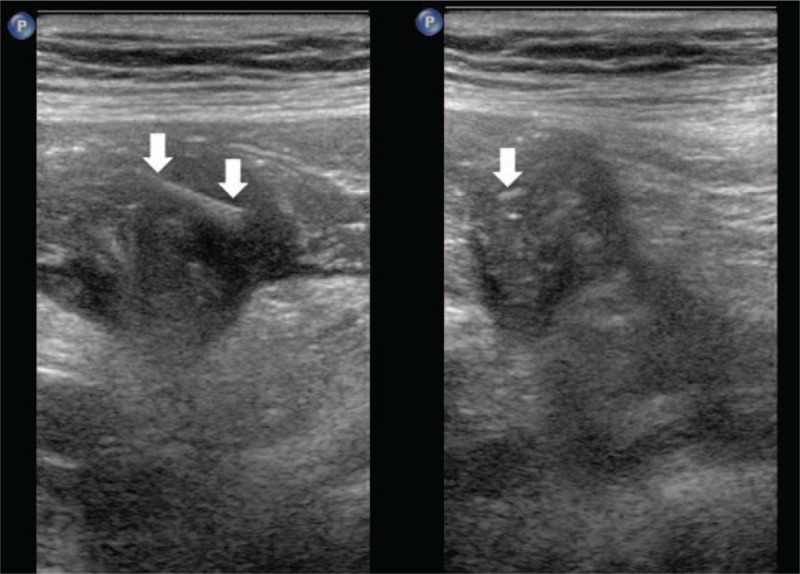
Fish bone perforating small intestinal on ultrasound images. An ingested foreign body (arrow) presumed to be a fish bone is incidentally seen in the small intestinal on these ultrasound images. Ultrasound images demonstrating a linear echogenic (short axis on the right, long axis on the left) foreign body (arrows).

Ultrasonography revealed a vague right lower quadrant irregularly shaped 40 mm × 23 mm × 40 mm hypoechoic area without clear borders. No obvious blood flow was identified in this region. There appeared to be lumped bowel loops with internal echogenic clutter. There was also moderate surrounding mesenteric edema. A hypoechoic area adjacent to the intestine was also seen measuring approximately 10 mm × 15 mm. This area contained an irregular flaky echo with comet tail artifact, suggestive of a foreign body (Fig. 1). Overall, the ultrasound findings suggested a diagnosis of right lower abdominal intestinal mass with surrounding inflammation and free fluid, and with small intestinal perforation not excluded. Ultrasound findings at our hospital led our suspicions away from appendicitis and to foreign body. Our hospital ultrasound physician repeatedly asked the patient for further history. Upon discovering that the patient experienced significant senility, our physician felt that the possibility of a foreign body was likely. A provisional diagnosis of small bowel perforation secondary to fish bone perforation was made.

**Figure 2:**
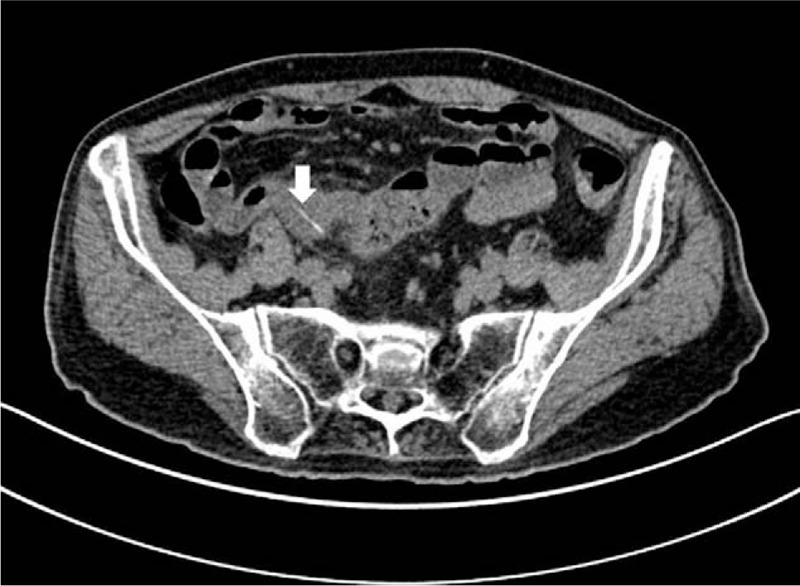
Fish bone perforating small intestinal on CT image. Axial noncontrast CT image demonstrating an intestinal foreign body (arrow) with associated small intestinal wall thickening and edema. The high-density foreign body with a diameter of 2.9 mm was found to represent aluminum pill packaging. Note also the surrounding mesenteric inflammatory fat stranding.

**Figure 3:**
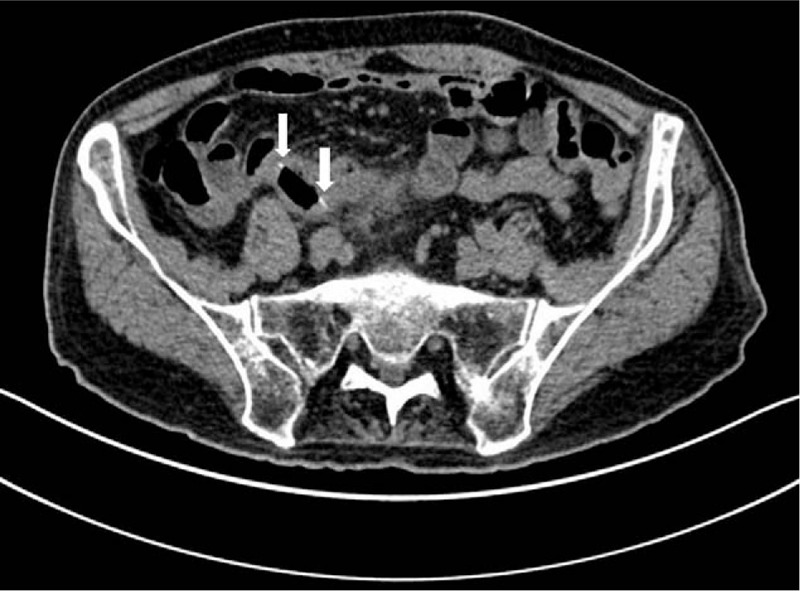
An additional axial CT image. An additional axial CT slice through the foreign body across the center of the pill packaging.

CT revealed intestinal swelling in the right lower abdomen, with a ring-shaped high-density structure measuring 2.9 mm, most consistent with a foreign body. There was blurring of the surrounding fat, suggestive of mesenteric edema as well as adjacent reactive mesenteric lymph nodes. There was no free air, though contained perforation was not excluded. Overall, the CT findings suggested a diagnosis of enteritis secondary to irritation by a foreign body (Figs. 2 and 3). As such the clinical diagnosis on admission was ileus secondary to foreign body.

## 5 Therapeutic intervention

**Figure 4:**
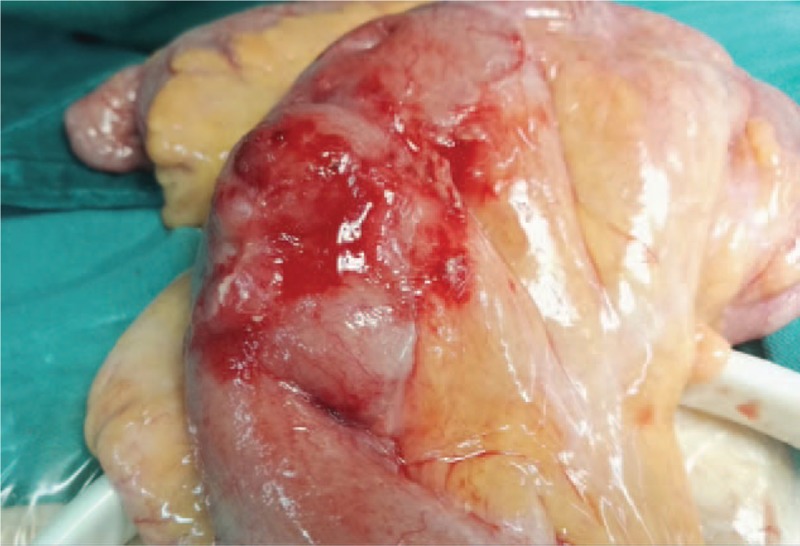
The intraoperative photograph. Intraoperative photograph of the ileum approximately 120 cm from the ileocecal valve. An approximately 2 mm perforation can be seen on the antimesenteric side of the ileum. This portion of the intestinal wall and mesentery were found to be swollen. Dark green liquid was expressed from the site of perforation. The foreign body was able to be palpated.

**Figure 5:**
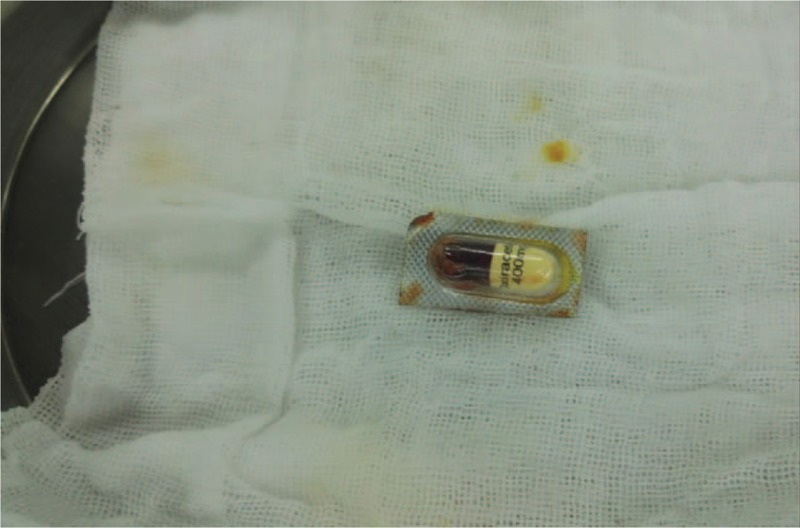
The foreign body. The foreign body (an oxiracetam pill still inside its aluminum-plastic synthetic packaging, measuring about 15 × 20 mm) was removed by expanding the ileal perforation site.

Emergency laparotomy under general anesthesia was performed. Intraoperatively, approximately 100 mL of light yellow clear liquid was identified in the abdomen and pelvis. Multiple membranous adhesions were noted adjacent to small bowel loops, though no bowel dilatation was evident. The bowel was examined and adhesions were removed. Approximately 10 mL of pus was obtained. Right lower quadrant intestinal wall and mesenteric edema were noted. There was small pus in the mesentery. On the anti-mesenteric side of the ileum, a 2 mm perforation was identified, which expressed dark green liquid. The site of perforation was expanded so that the foreign body could be removed. A capsule and packaging measuring 15 mm × 20 mm were obtained (Figs. 4 and 5). Furthermore, the tip of the appendix was noted to be swollen and edematous. As such, an appendectomy was performed.

## 6 Follow-up and outcomes

After the surgical procedure, the patient resumed anti-inflammatory treatment (Cefoxitin sodium 2000 mg tid) and rehydration therapy (Sodium Chloride Solution 100 mL tid). Further history obtained after surgery revealed that the patient had been being treated with oxiracetam pills for Alzheimer's disease. His most recent oral administration of the drug was 4 days prior, at which point the patient consumed the pill without removing the aluminum-plastic outer packaging. This history is obtained by the caregiver. The caregiver emphasized to the patient that he should make sure the packaging is removed before taking pills in the future. No follow-up diagnostics were necessary, and the patient was discharged without incident.

## 7 Discussion

Ultrasound because of its lack of ionizing radiation, convenience, and low cost is the preferred diagnostic imaging modality of the acute abdomen in China. Ultrasound findings related to small intestinal perforation caused by foreign bodies can be divided into direct signs and indirect signs. Direct signs include disruption of the intestinal wall and expression of intraluminal contents. However, with foreign bodies, intestinal perforations tend to be small, and thus the direct signs are difficult to see.^[5]^ Furthermore, if the perforation is self-contained, expression of intraluminal contents may not be seen.^[6]^ Therefore, when indirect signs including intestinal free air, free fluid, peristalsis, obstruction, twisting of bowel, and abscess are encountered, a differential diagnosis for the cause of these signs should include foreign body ingestion.^[7]^ A careful analysis of pertinent history should be performed before ultrasound examination of patients with an acute abdomen. During sonography, in addition to focusing on target areas and organs, surrounding adjacent structures should also be examined for abnormalities. These methods will help the sonographer identify secondary findings that may point to the diagnosis. When ultrasound imaging cannot obtain a diagnosis, other modalities such as abdominal X-ray, CT, or magnetic resonance imaging (MRI) may be necessary. CT has a high sensitivity and specificity for the detection of free air, and therefore for gastrointestinal perforation.^[8,9,10]^ Compared to MRI, CT is also faster. Compared to X-ray, CT is more sensitive, as it can demonstrate smaller amounts of extraluminal gas. As the abdominal cavity can be divided into different spaces, the location of gas can help point to the location and sometimes the etiology of the perforation.^[11,12]^ CT can also demonstrate discontinuity of the intestinal wall, the location of leakage of orally ingested contrast, the location of intestinal obstruction, the gas pattern within the intestine, thickening of the intestinal wall, associated masses or abscesses, and fistulas.^[11]^ Calcified vascular lesions and strangulated small bowel obstructions are also readily identified on CT. CT can also identify the foreign body itself.^[13]^ Although the foreign body is usually located at the site of perforation, occasionally, the foreign body may move distally from the site of perforation, so that the location of the foreign body does not necessarily coincide with the location of the perforation. The amount of abdominal or mediastinal free air varies depending on the degree and duration of the perforation.

## 8 Patient perspective

The patient did not share his perspective or experience.

## 9 Informed consent

The informed consent was obtained.

## 10 Conclusion

Foreign bodies tend to cause gastrointestinal perforation in young children, alcoholics, the elderly, and patients with mental disorders. These patients often cannot provide a detailed history, which can lead to misdiagnosis. Furthermore, if preoperative consideration is not sufficient, or the course of surgery is not meticulous enough (e.g., laparoscopic surgery or small lesions or foreign bodies), a second surgery may be necessary. Therefore, preoperative assessment should be thorough and a reasonable surgical approach should be chosen to avoid unnecessary pain and economic burden to patients.

## Author contributions

Gou Zehui wrote the manuscript. Gou Zehui and Yang Kun diagnosed and treated the patient. Gou Zehui performed acquisition of ultrasound data as well as interpreted the ultrasound images. All authors discussed the results of and edited the manuscript. Peng Yulan critically reviewed the manuscript.

**Conceptualization:** Ze-hui Gou.

**Data curation:** Ze-hui Gou.

**Formal analysis:** Ze-hui Gou.

**Writing – original draft:** Ze-hui Gou.

**Writing – review & editing:** Ze-hui Gou, Yulan Peng.

**Validation:** Kun Yang.
